# PRINCE-1: safety and efficacy of atazanavir powder and ritonavir liquid in HIV-1-infected antiretroviral-naïve and -experienced infants and children aged ≥3 months to <6 years

**DOI:** 10.7448/IAS.18.1.19467

**Published:** 2015-06-10

**Authors:** Renate Strehlau, Anamaria Pena Donati, Pedro Martinez Arce, Jurgen Lissens, Rong Yang, Sophie Biguenet, Daniela Cambilargiu, Hélène Hardy, Todd Correll

**Affiliations:** 1 Rahima Moosa Mother & Child Hospital, Johannesburg, South Africa;; 2 Hospital Sotero Del Rio, Santiago, Chile; 3 Hospital Civil Fray Antonio Alcade, Guadalajara, Mexico; 4 Bristol-Myers Squibb Research and Development, Braine L'Alleud, Belgium; 5 Bristol-Myers Squibb Research and Development, Wallingford, CT, USA; 6 AbbVie, North Chicago, IL, USA; 7 Bristol-Myers Squibb Research and Development, Buenos Aires, Argentina; 8 Bristol-Myers Squibb Research and Development, Princeton, NJ, USA

**Keywords:** atazanavir powder, ritonavir liquid, HIV-1 infection, antiretroviral therapy, paediatric formulations, infant, child

## Abstract

**Introduction:**

PRINCE-1 is an ongoing prospective, international, multicentre, nonrandomized, two-stage clinical trial assessing safety and efficacy of once-daily atazanavir (ATV) powder boosted with ritonavir (RTV) liquid plus optimized dual nucleoside reverse-transcriptase inhibitor (NRTI) background therapy in antiretroviral (ARV)-naïve and -experienced children with HIV-1 infection aged ≥3 months to <6 years.

**Methods:**

Children with HIV-1 infection without prior ATV exposure and with a screening HIV-1 RNA ≥1000 copies/mL were enrolled. The dosing of ATV powder, boosted with 80 mg RTV liquid, was based on three baseline weight bands (5 to <10 kg=150 mg, 10 to <15 kg=200 mg and 15 to <25 kg=250 mg).

**Results:**

Of the 56 treated patients, 46 completed 48 weeks of therapy, 67.9% were from Africa and 60.7% were ART-naïve. Median ages at baseline were 6, 35 and 55 months, and proportions with HIV-1 RNA >100,000 were 85.7, 52.6 and 25% in the three baseline weight bands, respectively. No unexpected safety events occurred and no deaths were reported. Over 48 weeks, upper respiratory tract infections, diarrhoea, vomiting and Grade 3 to 4 hyperbilirubinaemia occurred in 35.7, 35.7, 28.6, and 9.4% of patients, respectively; five patients (8.9%) discontinued due to adverse events (AEs); and 11 patients (19.6%) experienced serious adverse events. At Week 48, using a modified intent-to-treat analysis (two patients were excluded because they switched to ATV capsules before Week 48), 61.1 and 74.1% of patients overall had an HIV-1 RNA level <50 copies/mL and <400 copies/mL, respectively. Virologic suppression rates increased across the lowest to highest baseline weight bands (47.6, 68.4 and 71.4% had HIV-1 RNA <50 copies/mL, and 66.7, 73.7 and 85.7% had HIV-RNA <400 copies/mL, respectively) but did not differ meaningfully between ARV-naïve and -experienced patients. Overall, the median change from baseline in CD4 cell count was +363 cells/mm^3^, and the median change from baseline in CD4 percent was +7.5%.

**Conclusions:**

ATV powder boosted with RTV liquid once daily plus optimized dual NRTI background therapy was effective and well tolerated in this ART-naïve or -experienced paediatric population aged ≥3 months to <6 years. No unexpected safety findings compared with those from previous ATV paediatric and adult studies were identified.

## Introduction

Of the estimated 35.3 million people living with HIV-1 infection in 2012, 3.3 million were children aged <15 years [1]. HIV-positive infants frequently present with symptoms in the first year of life [2], and these infants are at higher risk of morbidity and mortality compared with older children with horizontally acquired HIV infection [3]. Combination antiretroviral therapy (cART) regimens reduce AIDS-related morbidity, disease progression and mortality in infants [4] and children [5] with HIV-1 infection, with many children now surviving into adulthood as a result of effective treatment [6–8]. In the United States, mortality in children and adolescents fell sharply after 1996, coincident with the introduction of cART regimens [9], and has stabilized since 2000 to a level around 0.5 to 0.8 deaths per 100 person-years [5].

World Health Organization (WHO) treatment guidelines for HIV-1-infected infants and children recommend a cART regimen containing lopinavir (LPV) boosted with ritonavir (RTV) as a first-line treatment option for those aged <3 years [7]. Similarly, US Department of Health and Human Services (DHHS) treatment guidelines for infants, children and adolescents recommend two nucleoside reverse transcriptase inhibitors (NRTIs) in combination with a protease inhibitor (PI) (usually boosted with RTV) or a non-nucleoside reverse transcriptase inhibitor (NNRTI) [10,11]. However, despite the progress in developing novel antiretroviral (ARV) therapies for infants and children with HIV-1 infection, there have been few clinical trials in this population to evaluate the efficacy, safety, pharmacokinetics and optimal dosage regimens, particularly in children aged ≤6 years. There have also been relatively few formulations developed for paediatric patients, and treatment options are limited [11].

Only a few ARV drugs are available as palatable formulations (e.g. liquids, minitab sprinkles or powders for reconstitution with water or for mixing with food) suitable for administration to children. PIs currently available as liquid formulations/reconstituted oral suspensions for the treatment of paediatric patients in the United States include fosamprenavir (aged 2 to 18 years) [12], coformulated RTV-boosted LPV (LPV/r) (aged ≥14 days) [13,14], nelfinavir (≥2 years) [15] and RTV-boosted darunavir (DRV/r) (aged 3 to 18 years) [11,16].

Atazanavir (ATV), an azapeptide PI of HIV-1, is indicated for the treatment of HIV-1 infection in combination with other ARV drugs in adults and children and is available in capsule (150 mg, 200 mg, 300 mg) and powder (50 mg) formulations [17]. The open-label, multicentre, Phase I/II PACTG 1020A (AI424-020/NCT00006604) study has shown that combination regimens, including ATV capsules boosted with RTV (ATV/r), are effective and well tolerated in children and adolescents aged ≥6 years [18]. Using data from PACTG 1020A, in which 176 paediatric patients aged 3 months to 21 years received ATV powder or capsules with or without RTV, and data from three adult studies, Hong *et al*. conducted model-based simulations to identify weight-based ATV doses that were predicted to produce ATV exposures in children similar to those achieved in adults receiving the recommended adult ATV/r dose of 300/100 mg [19].

Results from the Hong *et al*. modelling study were used to inform the weight-based dosing schedules evaluated in the ATV powder development program studies, PRINCE-1 (AI424-397/NCT01099579) and PRINCE-2 (AI424-451/NCT01335698). Data from the PRINCE studies, together with those from PACTG 1020A, have supported the recent US Food and Drug Administration (FDA) approval of ATV powder for use in paediatric patients ≥3 months of age and weighing ≥10 kg to <25 kg [20]. Here, we present the PRINCE-1 primary safety findings and secondary efficacy results (proportion achieving virologic suppression, reduction in HIV-1 RNA level and increase in CD4 count) over 48 weeks.

## Methods

### Design

This Phase IIIb, prospective, international, multicentre, nonrandomized, two-stage study was conducted in 14 centres across six countries (Brazil, Chile, Mexico, Peru, South Africa and Thailand) and enrolled a cohort of HIV-1-infected paediatric patients aged ≥3 months to <5.5 years.

The study was conducted in accordance with good clinical practice (GCP), as defined by the International Conference on Harmonisation (ICH), and in accordance with the ethical principles underlying European Union Directive 2001/20/EC and the US Code of Federal Regulations, Title 21, Part 50 (21CFR50). The study was approved by institutional review boards and independent ethics committees for the participating centers. Informed consent was obtained from all parents/legal guardians and assent from minors who were judged to be at an age of reason. An independent data monitoring committee (DMC) oversaw progress of the study.

### Patients

Patients were ARV-naïve or -experienced (without prior exposure to ATV) with screening plasma HIV-1 RNA levels of ≥1000 copies/mL. Treatment-experienced patients were defined as having had previous exposure to ARVs either as prior treatment for HIV-1 infection or as postnatal treatment with ≥1 ARV drug for the prevention of mother-to-child transmission. ARV-naïve patients had to show genotypic sensitivity to ATV and to both components of the local NRTI backbone at screening. ARV-experienced patients had to show genotypic and phenotypic sensitivity to ATV and two NRTIs at screening. NRTIs used were all required to have been approved for paediatric use at the local country level.

Key exclusion criteria were as follows. Children with previous exposure to ATV or ATV/RTV or prior history of two or more failures during PI treatment were excluded in order to assess the investigational formulation in ATV-naïve patients who were unlikely to have PI resistance. Children with documented cardiac conduction abnormality, significant cardiac dysfunction or a history of syncope were excluded on the basis that US treatment guidelines state that LPV/r must be used with caution in patients with pre-existing conduction system defects, because these agents can prolong the PR and QT intervals of the electrocardiogram (ECG) [11]. In addition, ATV is also known to affect the ECG [17] and the study was intended to identify the occurrence of potential drug-related cardiac conduction disorders. Children with coinfection with either hepatitis B virus or C virus were excluded in order to specifically evaluate the effect of the investigational formulation in those with HIV-1 infection only.

### Treatments

Backbone therapy consisted of two NRTIs that were approved for paediatric use and administered at doses in accordance with local treatment guidelines. The NRTI regimen was determined by the investigator on the basis of the viral resistance profile and treatment history. The use of tenofovir disoproxil fumarate (TDF) was excluded because of its known pharmacokinetic interaction with ATV (TDF decreases ATV trough concentration by 23% [17]). During Stage 1, eligible ARV-naïve and -experienced HIV-1-infected children between the ages of ≥3 months to <5.5 years with screening plasma HIV-RNA ≥1000 copies/mL received once-daily ATV powder formulation boosted with RTV liquid and two NRTIs (mainly administered twice daily). The dosing schedule for ATV powder and RTV liquid, which was determined by weight bands, is shown in Table 1. ATV powder is available as 50-mg sachets, which were preferably administered with a small amount of food or beverage (e.g. water, milk, infant formula, applesauce or yoghurt). If water was used, the mixture had to be taken with food. RTV liquid was administered immediately before or after ATV powder [21].

**Table 1:** Dosing schedule in children aged ≥3 months to <6 years for atazanavir power and ritonavir liquid by baseline weight bands

Body weight (kg)	ATV dose (mg)	RTV dose (mg)	Backbone
5 to <10	150	80	+ approved NRTI backbone
10 to <15	200	80	(tenofovir disoproxil fumarate
15 to <25	250	80	was prohibited)

ATV, atazanavir powder; RTV, ritonavir liquid.

Patients who completed 48 weeks on ATV powder transitioned to Stage 2 and continued taking ATV powder. When patients reached six years of age or 25 kg in weight at any time point through 48 weeks, they entered into Stage 2 and transitioned to treatment with ATV and RTV capsules. Data collection for Stage 2 is ongoing and will be reported subsequently.

### Outcomes

Assessments were performed at all visits (screening, Day 1 baseline and Weeks 4, 8, 12, 16, 24, 32, 40 and 48).

#### Primary outcome

The primary outcome was the safety of ATV powder formulation boosted with RTV liquid as part of a cART regimen in paediatric patients dosed through 48 weeks (or a minimum of 24 weeks for patients aged 5.5 years at the time of the study start). Safety endpoints included the following: the frequency of general adverse events (AEs), serious adverse events (SAEs), AEs considered related to study treatment and AEs leading to discontinuation of study treatment; Centers for Disease Control (CDC) Class C AIDS events (the 2008 classification system [22] was used as this was the system in place at the time of data collection and analysis, rather than the recently published 2014 classification [23]); and Grade 3 to 4 laboratory abnormalities, classified according to the Division of AIDS grading system [24], with the highest toxicity grade reported for each laboratory test.

#### Secondary outcomes

The efficacy of treatment with the ATV powder formulation was measured by the proportion of patients with virologic suppression, as defined by HIV-1 RNA <50 copies/mL or <400 copies/mL at Week 48, using the Roche Amplicor HIV-RNA Assay (version 1.5; Roche Diagnostic Systems, Inc., Branchburg, NJ, USA). Efficacy endpoints were summarized by baseline weight band (5 to <10 kg, 10 to <15 kg and 15 to <25 kg) and by prior ARV use (ARV-naïve or ARV-experienced patients).

Virologic suppression rates at Week 48 were analyzed using a modified intent-to-treat (ITT) approach where the numerator was based on the number of patients with on-treatment HIV-1 RNA values <50 (or <400) copies/mL at Week 48 and the denominator was based on the total number of patients in the ATV powder cohort (treated patients who did not switch to ATV capsule at or before Week 48).

Virologic suppression rates at Week 48 were also analyzed using observed values where the numerator was based on the number of patients with on-treatment HIV-1 RNA values <50 (or <400) c/mL at Week 48 and the denominator was based on patients with available HIV-1 RNA values at Week 48.

Other efficacy assessments included changes from baseline at Week 48 in HIV-1 RNA log_10_ values, CD4 cell count and CD4 percentage.

Viral genotypic and phenotypic resistance profiles were assessed in patients who experienced virologic failure and who met the criteria for resistance testing. The criteria for virologic failure were as follows: (1) a confirmed <1 log_10_ decrease from baseline in HIV-1 RNA levels by Week 16; or (2) a confirmed HIV-1 RNA level of >200 copies/mL after Week 24; or (3) repeated HIV-1 RNA levels of ≥50 copies/mL after Week 48; or (4) a confirmed HIV-1 RNA level of ≥400 copies/mL occurring at any time in a patient who had previously achieved an HIV-1 RNA level of <50 copies/mL. The criteria for resistance testing were as follows: (1) meeting criteria for virologic failure; or (2) discontinuing study treatment for any reason other than withdrawal of consent. HIV-1 isolates were tested for phenotypic resistance using the Phenosense™ HIV assay (Monogram Biosciences, Inc., South San Francisco, CA, USA). HIV substitutions were determined using the GeneSeq™ HIV assay (Monogram Biosciences, Inc.). Both assays request/require a viral load >400 copies/mL. Newly emergent genotypic substitutions were defined as on-treatment substitutions that were not detected at baseline. Newly emergent phenotypic resistance was defined as a baseline fold change less than the cut-off for reduced susceptibility and an on-treatment fold change greater than the cut-off for reduced susceptibility.

The acceptability and palatability of ATV powder and RTV liquid were assessed using a caregiver questionnaire administered at each study visit, in which caregivers were asked if their child had any trouble completing the dose of ATV powder or RTV liquid (acceptability). Reasons for having trouble completing the dose included dislike of taking medicines generally, specific dislike of the taste of the study medications (palatability) and regurgitation of the dose.

The pharmacokinetic profile of ATV powder formulation administered with RTV liquid was also evaluated in this study, as well as in the PRINCE-2 study. Given that the ongoing PRINCE-2 study evaluates a higher dose of ATV (200 mg) in the 5 to <10 kg baseline weight band, an additional baseline weight band of 25 to <35 kg and an age range of 6 to <11 years, none of which were represented in PRINCE-1, pharmacokinetic results from both studies will be presented in a future combined publication.

### Statistical analyses

Given that the primary outcome was the safety of the ATV powder formulation boosted with RTV liquid, formal statistical hypothesis testing was not planned and accordingly power calculations were not undertaken. However, a target sample size of 50 treated patients can detect, with 80% probability, a safety event that occurs at a per patient incident rate of 3.2%.

Descriptive summaries were presented for treated patients in total, categorized by baseline weight band (5 to <10 kg, 10 to <15 kg and 15 to <25 kg) or by prior ARV experience (ARV-naïve, ARV-experienced). No statistical comparisons between subgroups were conducted, and only summary statistics were presented. Categorical variables were summarized with counts and percentages or with proportions (number with event divided by number evaluable) expressed as percentages. For the percentages of patients with virologic suppression, exact binomial 95% confidence intervals (CI) were calculated. Continuous variables were summarized with univariate statistics. All analyses were conducted using the statistical software SAS^®^ version 9 (SAS Institute, Inc., Cary, NC, USA).

## Results

### Patients

Of 82 screened patients from 14 sites, 56 (68%) were successfully enrolled and treated (Table 2). Of these 56 treated patients, 46 (82%) completed Stage 1. Nine patients (16%) discontinued before Week 48, with the most common reason for discontinuation of ATV powder being an AE (five patients), particularly in the lowest weight band. One patient discontinued treatment at Week 48 due to poor/non-compliance. One patient withdrew consent and two patients discontinued due to lack of efficacy. At baseline, the majority of patients were ARV-naïve (60.7%). Median ages at baseline were 6, 35 and 55 months; proportions with HIV-1 RNA >100,000 were 85.7, 52.6 and 25%; and median CD4 counts were 1815, 1002 and 669 cells/mm^3^ in the three baseline weight bands, respectively (Table 2). Overall, the most frequent NRTI backbone, chosen according to local guidelines and the patient's viral resistance profile at screening, was abacavir+lamivudine.

**Table 2:** Patient disposition and baseline characteristics

	Body weight at baseline			
		
	5 to <10 kg (*N=*21)	10 to <15 kg (*N=*19)	15 to <25 kg (*N=*16)	Total (*N=*56)
Patient disposition				
Treated	21	19	16	56
Completed Stage 1 period (%)	17 (81)	14 (73.7)	15 (93.8)	46 (82.1)
Discontinued before Week 48	4 (19)	4 (21.1)	1 (6.3)	9 (16.1)
Adverse events	4 (19)	1 (5.3)	0	5 (8.9)
Withdrew consent	0	0	1 (6.3)	1 (1.8)
Lack of efficacy	0	2 (10.5)	0	2 (3.6)
Poor/noncompliance	0	1 (5.3)	0	1 (1.8)
Age in months, median (min–max)	6 (3–15)	35 (21–54)	55 (34–65)	28.5 (3–65)
Female, *n* (%)	10 (47.6)	12 (63.2)	6 (37.5)	28 (50)
Region (%)				
Africa	17 (81)	13 (68.4)	8 (50)	38 (67.9)
Asia	0	1 (5.3)	0	1 (1.8)
North America	2 (9.5)	3 (15.8)	4 (25)	9 (16.1)
South America	2 (9.5)	2 (10.5)	4 (25)	8 (14.3)
Weight in kg, median (min–max)	6.9 (5.4–9.3)	12.0 (10.7–14.8)	16.0 (15.0–21.1)	11.6 (5.4–21.1)
HIV-1 RNA log_10_ copies/mL, median (min–max)	5 (2.8–5)	5 (4–5)	4.28 (3.1–5)	5 (2.8–5)
HIV-1 RNA >100,000 copies/mL, *n* (%)	18 (85.7)	10 (52.6)	4 (25)	32 (57.1)
CD4 cells/mm^3^, median (min–max)	1815 (84–3451)	1002 (46–2172)	669 (106–1019)	1004 (46–3451)
CD4 percent categories (%)				
<15	2 (9.5)	2 (10.5)	1 (6.3)	5 (8.9)
15 to <25	7 (33.3)	6 (31.6)	4 (25)	17 (30.4)
≥25	7 (33.3)	6 (31.6)	6 (37.5)	19 (33.9)
Not reported	5 (23.8)	5 (26.3)	5 (31.3)	15 (26.8)
ARV-experienced patients	8 (38.1)	7 (36.8)	7 (43.8)	22 (39.3)
Prior ARVs received				
Abacavir	1/8 (12.5)	0	2/7 (28.6)	3/22 (13.6)
Didanosine	0	1/7 (14.3)	0	1/22 (4.5)
Efavirenz	0	1/7 (14.3)	3/7 (42.9)	4/22 (18.2)
Emtricitabine	0	0	1/7 (14.3)	1/22 (4.5)
Lamivudine	1/8 (12.5)	4/7 (57.1)	6/7 (85.7)	11/22 (50.0)
Lopinavir/ritonavir	1/8 (12.5)	4/7 (57.1)	4/7 (57.1)	9/22 (40.9)
Nevirapine	7/8 (87.5)	2/7 (28.6)	2/7 (28.6)	11/22 (50.0)
Ritonavir	0	1/7 (14.3)	0	1/22 (4.5)
Stavudine	0	3/7 (42.9)	4/7 (57.1)	7/22 (31.8)
Zidovudine	1/8 (12.5)	3/7 (42.9)	2/7 (28.6)	6/22 (27.3)
Backbone NRTIs administered				
Abacavir	17 (81.0)	16 (84.2)	13 (81.3)	46 (82.1)
Didanosine	0 (0)	2 (10.5)	0 (0)	2 (3.6)
Lamivudine	20 (95.2)	12 (63.2)	9 (56.3)	41 (73.2)
Stavudine	0 (0)	1 (5.3)	3 (18.8)	4 (7.1)
Zidovudine	5 (23.8)	6 (31.6)	10 (62.5)	21 (37.5)

## Safety

### General AEs

No deaths were reported. Eleven patients (19.6%) treated with ATV powder had on-treatment SAEs. Herpes zoster was the only SAE reported in two or more patients (3.6%), occurring in two patients, and was considered unrelated to study therapy by the investigator.

Five patients (8.9%) had AEs leading to discontinuation of study therapy: four in the 5 to <10 kg group (meningitis [*n=*1] and pulmonary tuberculosis [*n=*1] considered unrelated to study therapy by the investigator; increased transaminases [*n=*1] and lymphadenitis [*n=*1] considered as related events) and 1 in the 10 to <15 kg group (prolonged QT interval that was also considered an SAE). The patient discontinuing ATV due to increased transaminases had a history of nevirapine-induced hepatitis and was receiving concomitant hydroxyzine, an agent known to cause liver dysfunction [25]. In this patient, transaminase measurements returned to normal 30 days after discontinuation of study therapy without the need for specific treatment. In the patient with QT prolongation, a Fridericia's corrected QT (QTcF) interval of 477 msec was observed, on a protocol-scheduled ECG, on Day 163 compared with a baseline value of 382 msec. The study investigator considered the QT prolongation to be related to study therapy, which was discontinued on Day 170. However, the patient was also receiving concomitant metronidazole and sulfamethoxazole-trimethoprim, both of which have been reported to cause QT prolongation [26,27]. Repeated ECGs after study drug discontinuation showed a normal QT interval without the need for specific treatment.

Through Week 48, the majority of patients on ATV powder (92.9%) had at least one AE, with the most common being upper respiratory tract infection, diarrhoea and vomiting (Table 3). The most common AEs considered related to study therapy by the investigator were hyperbilirubinaemia (seven patients, 12.5%) and vomiting (five patients, 8.9%). Among the seven patients with AEs of hyperbilirubinaemia, two also had AEs of clinical jaundice, but none of these patients discontinued study therapy due to hyperbilirubinaemia.

**Table 3:** Adverse events and Grade 3 to 4 laboratory abnormalities in patients receiving ATV powder through Week 48

	Body weight at baseline			
		
	5 to <10 kg (*N=*21)	10 to <15 kg (*N=*19)	15 to <25 kg (*N=*16)	Total (*N=*56)
**Adverse events of any grade occurring with a frequency of at least 20% in any group**				
Total patients with event, *n* (%)	20 (95.2)	18 (94.7)	14 (87.5)	52 (92.9)
Infections	18 (85.7)	16 (84.2)	12 (75.0)	46 (82.1)
Upper respiratory tract infection	11 (52.4)	5 (26.3)	4 (25.0)	20 (35.7)
Nasopharyngitis	4 (19.0)	3 (15.8)	5 (31.3)	12 (21.4)
Otitis media	6 (28.6)	3 (15.8)	1 (6.3)	10 (17.9)
Gastrointestinal disorders	13 (61.9)	11 (57.9)	8 (50.0)	32 (57.1)
Diarrhoea	11 (52.4)	6 (31.6)	3 (18.8)	20 (35.7)
Vomiting	6 (28.6)	6 (31.6)	4 (25.0)	16 (28.6)
Respiratory, thoracic and mediastinal disorders	11 (52.4)	7 (36.8)	7 (43.8)	25 (44.6)
Cough	5 (23.8)	5 (26.3)	4 (25.0)	14 (25.0)
Skin and subcutaneous disorders	14 (66.7)	4 (21.1)	6 (37.5)	24 (42.9)
Eczema	7 (33.3)	2 (10.5)	1 (6.3)	10 (17.9)
Diaper dermatitis	6 (28.6)	1 (5.3)	0	7 (12.5)
Blood and lymphatic system disorders	9 (42.9)	5 (26.3)	5 (31.3)	19 (33.9)
Neutropenia	5 (23.8)	1 (5.3)	0 (0)	6 (10.7)
**Grade 3 to 4 laboratory abnormalities^a^, *n* (%)**				
Haematology				
Haemoglobin	2/20 (10.0)	3/17 (17.6)	0/15 (0)	5/52 (9.6)
Neutrophils (absolute)	3/20 (15.0)	2/17 (11.8)	0/15 (0)	5/52 (9.6)
Liver function tests				
ALT/SGPT	5/20 (25.0)	0/18 (0)	1/15 (6.7)	6/53 (11.3)
AST/SGOT	1/20 (5.0)	0/18 (0)	0/15 (0)	1/53 (1.9)
Total bilirubin	2/20 (10.0)	0/18 (0)	3/15 (20.0)	5/53 (9.4)
Serum chemistries				
Total amylase	8/20 (40.0)	5/18 (27.8)	1/15 (6.7)	14/53 (26.4)
Lipase	0/20 (0)	1/18 (5.6)	1/15 (6.7)	2/53 (3.8)

ALT, alanine transaminase; AST, aspartate transaminase; SGOT, serum glutamic-oxaloacetic transaminase; SGPT, serum glutamic pyruvic transaminase; *N*, total number of patients; *n*, number of patients with each event.

a According to the Division of AIDS Table for Grading the Severity of Adult and Pediatric Adverse Events Version 1.0, December, 2004; Clarification August 2009 [24].

### Other significant AEs

Three patients (5.4%) had cardiac disorders. Two of these events were considered related to the study therapy by the investigator: ECG QT prolongation in one patient in the 10 to <15 kg group (described above in the section on AEs leading to discontinuation) and first-degree atrioventricular block in one patient in the 15 to <25 kg group. Eight patients (14.3%) had rash events, none of which were considered related to the study therapy by the investigator. One patient (1.8%) in the 5 to <10 kg group had pulmonary tuberculosis, a CDC Class C AIDS event [22], which was considered unrelated to study therapy by the investigator. Two patients (3.6%) had renal toxicity events, both considered unrelated to study therapy by the investigator (proteinuria in one patient in the 5 to <10 kg group and dysuria in one patient in the 10 to <15 kg group). None of the patients had lipodystrophy-related events.

### Laboratory abnormalities

The most common Grade 3 to 4 laboratory abnormality was elevated total serum amylase (26.4%); however, all of these patients had normal fractionated pancreatic amylase levels. Grade 3 to 4 elevations in total bilirubin were present in 5/53 (9.4%) of patients and appeared not to systematically vary by baseline weight band. Grade 3 to 4 elevations in serum transaminases were present in 7/53 (13.2%) of patients, but no instance of drug-induced liver injury was reported. Other Grade 3 to 4 laboratory abnormalities are summarized in Table 3.

## Efficacy

**Figure 1:**
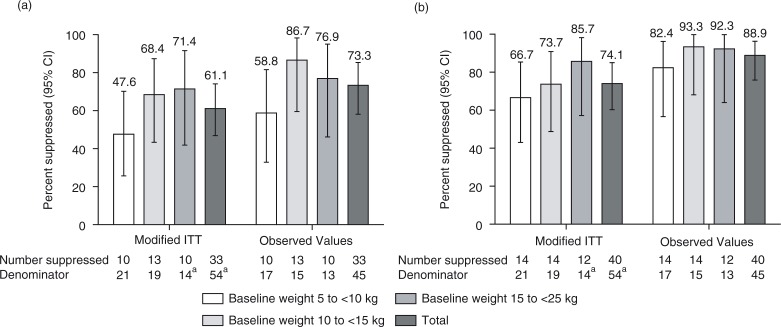
Percentage of patients achieving virologic suppression by baseline weight bands at Week 48: (a) HIV-1 RNA <50 copies/mL and (b) HIV-1 RNA <400 copies/mL. ITT, intent-to-treat. ^a^Two patients were not included in this analysis because they had switched to ATV capsules before Week 48. Data are proportions expressed as percentages with exact binomial 95% confidence intervals (CI).

Using a modified ITT approach based on the Week 48 ATV powder cohort (patients who did not switch to the ATV capsule at or before Week 48), 61.1% of patients had HIV-1 RNA levels of <50 copies/mL and 74.1% had HIV-1 RNA levels of <400 copies/mL. Virologic suppression rates increased progressively across the lowest to highest baseline weight bands (Figure 1). Virologic suppression rates were generally higher across all baseline weight bands in the analysis using observed values; however, similar to the modified ITT analysis, rates in the lowest weight band were lower than those in the two higher weight bands for both HIV-1 RNA <50 and <400 copies/mL thresholds (Figure 1).

**Figure 2:**
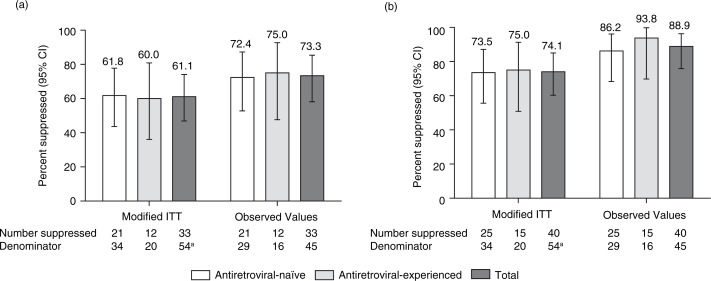
Percentage of patients achieving virologic suppression by prior antiretroviral experience at Week 48: (a) HIV-1 RNA <50 copies/mL and (b) HIV-1 RNA <400 copies/mL. ITT, intent-to-treat. ^a^Two patients were not included in this analysis because they had switched to ATV capsules before Week 48. Data are proportions expressed as percentages with exact binomial 95% confidence intervals (CI).

Virologic suppression rates showed no clear numeric differences between ARV-naïve and -experienced patients (Figure 2).

**Figure 3:**
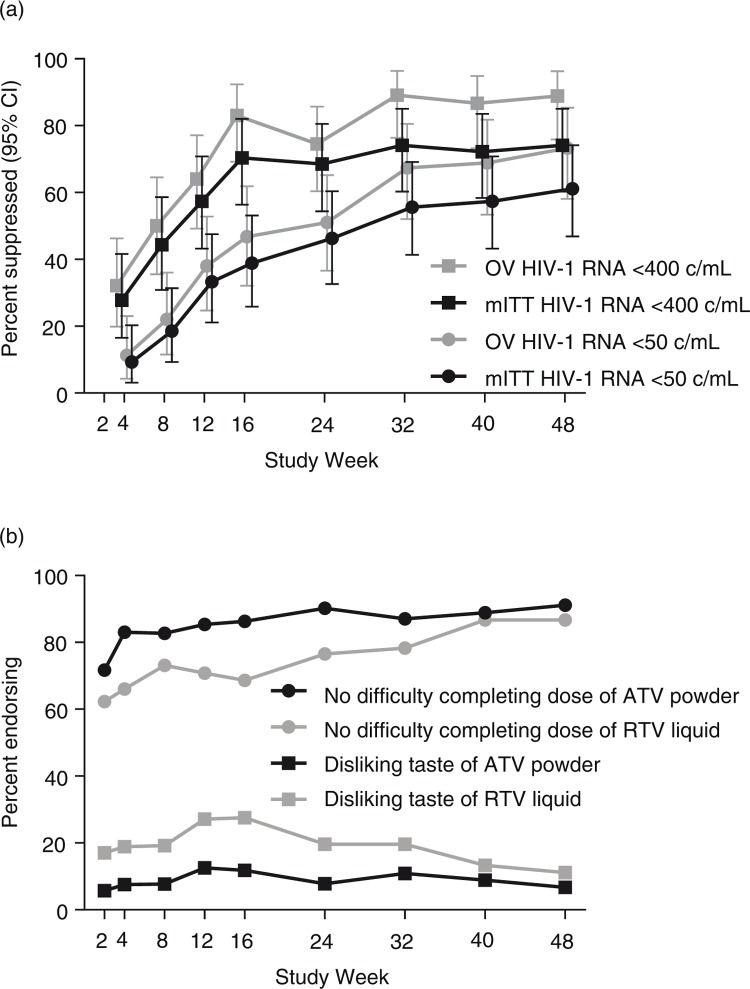
(a) Virologic suppression over time and (b) acceptability and palatability of ATV powder and RTV liquid. mITT, modified intent-to-treat; OV, observed values.

Virologic suppression rates improved progressively over time for all patients (Figure 3a), regardless of method of analysis (modified ITT vs. observed values) or threshold HIV-1 RNA level (<50 copies/mL vs. <400 copies/mL).

At Week 48, the overall mean change from baseline in HIV-1 RNA levels was −2.66 log_10_ copies/mL (−2.61, −2.93and −2.40 log_10_ copies/mL in the 5 to <10 kg, 10 to <15 kg and 15 to <25 kg groups, respectively).

The acceptability of ATV powder was higher than that for RTV liquid over the first 24 weeks. Drug acceptability tended to improve over time for both treatments (Figure 3b) but remained slightly higher for ATV powder at Week 48 (the majority of patients had no trouble completing their doses; 91.1 and 86.7% for ATV powder and RTV liquid, respectively). ATV powder was more palatable than RTV liquid over the first 24 weeks. Drug palatability tended to improve after 16 weeks for both treatments (Figure 3b), but ATV powder remained more palatable than RTV liquid at Week 48 (6.7 and 11.1% of patients disliked the taste of ATV powder and RTV liquid, respectively). Most caregivers mixed ATV powder with water (48.9%) or milk (28.9%). There were no numeric differences between the lowest baseline weight band and the two higher baseline weight bands combined in rates of acceptability at Week 48 for ATV powder (94.1% vs. 89.3%, respectively) or RTV liquid (88.2 and 85.7%, respectively). Similarly, there were no numeric differences between the lowest baseline weight band and the two higher baseline weight bands combined in rates of dislike of the taste at Week 48 for ATV powder (5.9% vs. 7.1%, respectively) or RTV liquid (11.8 and 10.7%, respectively).

Immunologic recovery was observed across all baseline weight bands, with a trend to greater changes from baseline in CD4 percent in the higher versus the lower baseline weight bands (Table 4). Despite baseline CD4 cell count levels being comparable between the ARV-naïve and -experienced groups, changes from baseline in CD4 cell count and CD4 percent were lower in ARV-experienced patients compared with ARV-naïve patients at Week 48 (Table 4).

**Table 4:** Immunologic recovery by baseline weight bands and prior antiretroviral experience at Week 48

		Baseline	Change from baseline at Week 48			
			
	*N*	Mean (median) CD4 cell count	*n*1	Mean (median) CD4 cell count	*n*2	Mean (median) CD4 percent
Baseline weight 5 to <10 kg	16	1594 (1815)	13	550 (491)	14	6.1% (6.0%)
Baseline weight 10 to <15 kg	13	1107 (1002)	11	225 (274)	12	7.3% (7.5%)
Baseline weight 15 to <25 kg	10	661 (669)	5	374 (363)	6	8.8% (9.5%)
ARV-naïve	25	1202 (1019)	20	493 (520)	22	8.8% (9.0%)
ARV-experienced	14	1175 (970)	9	182 (213)	10	3.2% (2.5%)
Total	39	1193 (1004)	29	397 (363)	32	7.0% (7.5%)

*N*, the number of patients with available baseline CD4 values; *n*1, the number of patients with available baseline and Week 48 CD4 cell count values; *n*2, the number of patients with available baseline and Week 48 CD4 percent values; ARV, antiretroviral.

### Resistance data

By Week 48, 14 of 46 (30.4%) patients met the criteria for virologic failure. Of these 14 patients, six (42.9%) were ARV-experienced and eight (57.1%) were ARV-naïve. Nine patients had paired genotypic resistance data (baseline and on-treatment data) and six had paired phenotypic data. None of the patients acquired phenotypic resistance to ATV, ATV/RTV or any NRTI or NNRTI. One patient developed phenotypic resistance to saquinavir. None of the patients developed any major PI substitution to ATV or ATV/RTV.

## Discussion

ATV powder boosted with liquid RTV in combination with optimized NRTI background therapy in infants and children aged three months to six years with HIV-1 infection was generally safe and well tolerated, with a safety profile similar to that reported using ATV capsules in adult studies [28–30] and in paediatric patients aged ≥6 years [18]. No new safety findings were identified that have not been previously reported in the aforementioned studies. Although not considered related to study therapy at the time of documentation, it is possible that the two instances of herpes zoster infection (one commencing on Day 14 and the other on Day 23) may have been caused by immune reconstitution inflammatory syndrome (IRIS), as they occurred within the 2- to 31-week window of onset for IRIS [31]. Therefore, these infections may have been indirectly related to study therapy. Similarly, the case of lymphadenitis, which occurred on Day 115 and was considered related to study therapy, may have been caused by IRIS. However, it should be noted that AEs in this study were extensively documented by study investigators and none were considered to be due to IRIS at the time of documentation. Therefore, it is not possible to attribute IRIS as a definitive cause for these AEs.

Virologic suppression (up to 73.3% with HIV-1 RNA <50 copies/mL and up to 88.9% with HIV-1 RNA <400 copies/mL, observed values) and immunologic response (median CD4 percent increase from baseline, 7.5%) at Week 48 was observed across all three baseline weight bands, regardless of prior ARV treatment experience. Results for the 10 to <15 kg and 15 to <25 kg baseline weight bands were consistent with results obtained in the PACTG 1020A trial using ATV capsules boosted with RTV in ARV-naïve patients aged 6 to <18 years with HIV-1 infection, for whom HIV-1 RNA levels of <50 or <400 copies/mL at Week 48 were achieved by 81 and 88% of patients, respectively [18].

Virologic suppression rate was lower in the 5 to <10 kg baseline weight band, which most likely resulted from the higher proportion of patients with a baseline plasma HIV-1 RNA level >100,000 seen in this group (85.7% compared with 52.6 and 25.0% in the 10 to <15 kg and 15 to <25 kg groups, respectively). A high plasma HIV-1 RNA level is commonly seen in infancy (the median age of patients in the 5 to <10 kg baseline weight band was six months), which, together with immunological immaturity, makes virologic suppression in infants more difficult to achieve and results in longer times to reach undetectable levels [32,33].

Potential alternative explanations for the lower virologic suppression rate in the 5 to <10 kg baseline weight band, such as the effect of prior ARV exposure, emergent resistance, differences in discontinuation rate or differences in acceptability/palatability were not supported by the available evidence. We found no difference in virologic suppression between ARV-naïve and -experienced patients. None of the patients who underwent resistance testing for virologic failure or discontinuation from the study acquired phenotypic resistance to ATV, ATV/RTV or any NRTI or NNRTI, and no major mutations associated with ATV resistance were reported. Discontinuation rates were not numerically different between the 5 to <10 kg and 10 to <15 kg baseline weight bands and, when analyzed using observed values, virologic suppression rates in the 5 to <10 kg baseline weight band remained numerically lower than those in the other baseline weight bands. Patients in the lowest baseline weight band had neither more trouble completing their doses, nor a greater dislike of the taste of study treatments compared with patients in the higher baseline weight bands. Whether pharmacokinetic differences could explain the lower virological suppression rate in the 5 to <10 kg baseline weight, especially because drug metabolism is known to differ substantially in infancy [34], will be fully explored in a forthcoming publication when these data are available.

Although making comparisons with paediatric clinical studies of other PIs is difficult owing to differences in trial design and endpoint definitions, the results of the current study are consistent with those from studies evaluating LPV/r and other PIs in paediatric populations. In the original study in children aged 6 months to 12 years with HIV-1 infection evaluating LPV/r, either added to stavudine and lamivudine (ARV-naïve) or nevirapine plus one or two NRTIs (ARV-experienced), overall, 79% had HIV-1 RNA levels <400 copies/mL at Week 48 using an ITT analysis. Similarly, in the PENPACT-1 trial, rates of virologic suppression with PIs (mainly LPV/r or nelfinavir) plus two NRTIs at Week 24 were 73% using an ITT analysis [35]. The more recent randomized P1060 trial conducted in resource-limited settings evaluated initial treatment with zidovudine, lamivudine and either nevirapine or LPV/r in children aged 2 to 36 months with HIV-1 infection without prior exposure to nevirapine. In this trial, virologic failure (a confirmed plasma HIV-1 RNA level <1 log_10_ copies/mL below the baseline level at 12 to 24 weeks after treatment initiation or a confirmed plasma HIV-1 RNA level of >400 copies/mL at 24 weeks) or treatment discontinuation occurred in 19.3% of patients randomized to LPV/r [36]. In patients with prior exposure to nevirapine, virologic failure or treatment discontinuation with LPV/r occurred in a slightly higher proportion (21.7%) compared with patients without prior exposure to nevirapine [37]. Recent studies evaluating fosamprenavir liquid boosted with RTV liquid have shown proportions achieving HIV-1 RNA levels <400 copies/mL (snapshot analyses) of 58% in patients aged 4 weeks to <6 months [38], 65% in patients aged 4 weeks to <2 years [38] and 74% in patients aged 2 years to <6 years [39]. Data on the use of DRV/r in children with HIV-1 infection are limited to ARV-experienced patients aged 6 to 17 years, of whom 59% achieved an HIV-1 RNA level <400 copies/mL after 48 weeks of treatment [40].

The acceptability of ATV powder boosted with RTV liquid was high among caregivers of the children in this study, with acceptability over 48 weeks ranging from 71.7 to 91.1% with ATV powder and from 62.3 to 86.7% with RTV liquid. The proportion of children reported by their caregivers as disliking the taste of ATV powder boosted with RTV liquid was low, with up to 12.5% disliking the taste of ATV powder over 48 weeks and up to 27.5% disliking the taste of RTV liquid over 48 weeks. These results compare favourably with the recently evaluated minitab sprinkle formulation of LPV/r, for which 38 to 53% of children <4 years of age were reported by their caregivers to have disliked the taste of the sprinkles [14].

Taken together, these findings on efficacy, safety and acceptability/palatability indicate that a regimen including ATV powder boosted with RTV liquid represents a new treatment option for infants and children aged ≥3 months to <6 years with HIV-1 infection. Indeed, data for this study have contributed to the recent FDA approval of ATV powder formulation for use for use in paediatric patients with HIV-1 infection ≥3 months of age and weighing ≥10 kg to <25 kg [20].

This study has limitations. The study enrolled a relatively small number of patients with HIV-1 infection, was non-randomized, and no statistical comparisons between subgroups were performed (by baseline weight band or ARV treatment experience). Numerically, virologic suppression rates were lower in patients in the lowest baseline weight band compared with the higher weight bands, and immunological recovery was less evident in ARV-experienced patients compared with ARV-naïve patients. However, the study design and small sample size did not allow these observations to be statistically verified. Over 48 weeks, up to 27.5% of patients were reported by their caregivers to have disliked the taste of RTV liquid. This percentage is lower than in a previous study assessing ARV adherence and palatability in children, of whom 50% disliked the taste of RTV liquid [41]. It should be noted that the palatability survey employed in our study was based upon caregiver report and may have underestimated the extent of children's dislike of the taste of RTV.

In conclusion, no new safety findings were identified that had not been previously reported in other ATV paediatric or adult studies. ATV powder boosted with liquid RTV in combination with optimized NRTI background therapy achieved adequate virologic suppression and immunologic response, was highly acceptable to caregivers, and was generally safe and well tolerated by infants and children aged three months to six years, regardless of prior ARV treatment experience or baseline weight band. No major PI substitutions associated with ATV resistance were reported. As recognized by the recent FDA approval, ATV powder is an available treatment option for use in paediatric patients with HIV-1 infection ≥3 months of age and weighing ≥10 kg to <25 kg.
